# Family Dynamics and Digital Distractions: A Survey-Based Study on How Co-Parenting and Parental Phubbing Shape Preschoolers’ Media Use

**DOI:** 10.3390/bs15060752

**Published:** 2025-05-30

**Authors:** Yuying Zhang, Kuai Song, Gengfeng Niu

**Affiliations:** 1 Key Laboratory of Adolescent Cyberpsychology and Behavior (CCNU), Ministry of Education, Wuhan 430079, China; zhangyy0528@mails.ccnu.edu.cn (Y.Z.); songpsy@mails.ccnu.edu.cn (K.S.); 2 Key Laboratory of Human Development and Mental Health of Hubei Province, School of Psychology, Central China Normal University, Wuhan 430079, China; 3 Center for Research on Internet Literacy and Behavior, Central China Normal University, Wuhan 430079, China; 4 Faculty of Preschool Education, Hubei Preschool Teachers College, Ezhou 436032, China

**Keywords:** co-parenting, parental phubbing, secure attachment, problematic media use, preschoolers

## Abstract

In the current information era, even preschool children are unable to withstand the “digital flood”. However, excessive exposure to electronic screens not only negatively impacts various aspects of children’s health and adaptation, but also harms family relationships. Based on family systems theory, social–cognitive learning theory, and attachment theory, this study examines the relationships between co-parenting and preschoolers’ problematic media use, as well as the underlying mechanism—the mediating role of parental phubbing and the moderating effect of secure attachment. A sample of 610 parents of preschoolers from three kindergartens in central China completed validated scales, including the Co-Parenting Scale, Parental Phubbing Scale, Children’s Electronic Media Use Questionnaire, and Secure Attachment Dimension of the Waters Attachment Q-sort. A moderated mediation model was tested using the PROCESS macro with bootstrap procedures. The results showed that, after controlling for the subjective family socioeconomic status and parental education level, (1) co-parenting was negatively associated with preschoolers’ problematic media use; (2) parental phubbing significantly mediated the relationship between co-parenting and preschoolers’ problematic media use; (3) secure attachment significantly moderated both the direct relation between co-parenting and the preschoolers’ problematic media use and the mediating effect of parental phubbing (the relation between parental phubbing and children’s problematic media use); furthermore, both of these effects were more pronounced in children with lower levels of secure attachment. These findings extend family systems and attachment theories by elucidating mechanisms underlying early media behaviors. Practically, interventions should promote collaborative co-parenting and reduce parental phubbing to mitigate children’s problematic media use, while fostering secure attachment to buffer digital risks.

## 1. Introduction

In the current information age, children are accessing the internet at increasingly younger ages. As “digital natives”, preschoolers are being introduced to, utilizing, or even owning electronic devices such as smartphones and tablet computers at an early age. According to a survey conducted by Guangming Daily (2023), 70.4% of preschoolers in China have begun interacting with and actively using mobile devices such as smartphones; and a substantial portion of these internet-engaged children possess their own dedicated devices. While these devices bring pleasure and convenience, they also expose young users to a range of risks including diminished eyesight, exposure to inappropriate content, sleep disturbances, and internet dependence or problematic internet use (Anis et al., 2020; Hisler et al., 2020; Muller, 2023). Problematic media use, distinct from general internet use, reflects excessive or dysregulated engagement that disrupts developmental outcomes (Xiang et al., 2022). Thus, problematic media use has become a critical focus due to its pervasive role in early development and its connection to internet access. Also, researchers have identified the negative impacts of problematic media use on preschoolers: poorer emotional functioning, which is detrimental to individuals’ psychological health, as well as behavioral problems related to delayed eating, toileting, and sleeping patterns (Xiang et al., 2022; Yalçin et al., 2021). Furthermore, for preschoolers, excessive exposure to electronic media may lead to deficits in language development, as these activities can impede interaction between toddlers and adults (Guellai et al., 2022).

Thus, preschoolers’ problematic media use represents an emerging developmental risk in the digital era, with its adverse effects well-documented. Against this background, the influencing factors and mechanism should be further examined, so as to provide evidence for scientific prevention and intervention. While some studies have incorporated multiple family-level variables or parental dyads, relatively few have systematically applied a family systems perspective to understand how interconnected parenting subsystems influence children’s digital behaviors (e.g., Cost et al., 2020; Niu et al., 2020). In particular, the co-parenting subsystem—defined as the collaborative process by which caregivers coordinate and support each other in child-rearing—remains underexplored in the context of media regulation (Kopko, 2007). Parental inconsistency in media rules (e.g., conflicting permissions from each parent) may contribute to increased child confusion and dysregulation, yet this issue has received limited empirical attention (Kildare & Middlemiss, 2017; McDaniel & Radesky, 2018). Moreover, many traditional frameworks have yet to account for emerging family-level risks in the digital age, such as parental phubbing—a behavior that not only models excessive media use but may also initiate a cycle of attention deprivation and compensatory digital engagement in young children (Radesky et al., 2018). Crucially, preschoolers respond differently to parental behaviors. Secure attachment may serve as a key protective factor, potentially moderating family influences through enhanced emotion regulation and stress coping capacities (Bowlby, 1969). Grounded in family systems theory and attachment theory (Bowen, 1993; Bowlby, 1969, 1979), we propose a dual-level (family–individual) model to investigate how co-parenting influences preschoolers’ problematic media use, with parental phubbing as a mediator and secure attachment moderating both the direct effect of co-parenting and the indirect effect of phubbing by buffering negative impacts.

### 1.1. Co-Parenting and Problematic Media Use Among Preschoolers

As a positive style of parenting, the importance of parental co-parenting has received a lot of attention across disciplines, as it benefits children of all ages (Eira Nunes et al., 2021). Co-parenting is an endeavor initiated by parents who are jointly responsible for the care and upbringing of children, and is concerned with the level of cooperation, affirmation, and support between parents who are raising children together (McHale, 2007). Co-parenting refers to coordinated interactions between caregivers regarding child-rearing practices, particularly in the domains of education and socioemotional development. This method involves coordination and mutual support, aiming to provide a unified approach to child-rearing (Feinberg, 2003; C. Liu et al., 2014). Co-parenting involves more than just sharing the labor or responsibilities of caring for a child; it also focuses on the consistency or degree of cooperation between parents in raising their child. Thus, parents can effectively function in the joint upbringing of their child, and thus help to foster a secure and stable environment for the child’s development and health (McHale, 2007). In addition, a co-parenting or a joint parenting alliance implies a focus on the active relationship between the child and the parents (Loper et al., 2014). Effective co-parenting provides a consistent and supportive caregiving environment, which fosters children’s emotional security, behavioral regulation, and social competence (X. Liu et al., 2024).

High-quality co-parenting, characterized by cooperation and consistent rule-setting, enhances family cohesion and communication, fostering adaptive child development and reducing problem behaviors, including excessive media use (Varga & Gee, 2017). When parents present a united front, communicate effectively, and support each other’s parenting efforts, children are more likely to experience stable boundaries and coherent expectations across caregivers. In addition, cumulative research demonstrates that positive, harmonious, and consistent parenting practices promote child development, parenting quality, and family mental health—including promoting children’s prosocial behaviors and improving marital relationships (Eira Nunes et al., 2021; Khawaja et al., 2024). Conversely, poor co-parenting, often linked to marital conflict and a disrupted family atmosphere, exacerbates child problem behaviors, including problematic media use (Qiao et al., 2024). Parental disagreement and conflict over media rules predicted higher problematic media use and socioemotional issues in young children, as inconsistencies allowed children to exploit lax rules, while conflict heightened family stress (Domoff et al., 2020). For example, when one parent restricts screen time while the other allows unrestricted access, children may receive inconsistent signals, undermining parental authority and increasing media use confusion. Another important study indicates that greater parental conflict over media rules is associated with increased child exposure to media violence, which, in turn, predicts higher levels of physical and relational aggression; conversely, when both parents strictly limit media use, exposure to media violence and parent–child conflict are reduced. Interestingly, rule disparities and related conflicts do not vary significantly by the child’s age (Mares et al., 2018). Although no studies have directly examined co-parenting’s link with preschoolers’ screen exposure or media dependence, the above explanations suggest that co-parenting quality may be significantly negatively associated with problematic media use.

### 1.2. The Mediating Role of Parental Phubbing

Parental phubbing, a prevalent behavior in this information era, refers to ignoring others due to smartphone fixation—constituting a unique form of social ostracism (Kang et al., 2023), which is common among many families. This behavior conveys emotional neglect, eroding parent–child intimacy, trust, and communication quality while heightening children’s loneliness (P. Wang et al., 2022). Social learning theory (Bandura & Walters, 1977) posits that children imitate parental behaviors, suggesting phubbing may model and reinforce excessive child media use. If children frequently observe their parents scrolling on smartphones during meals or playtime, they may imitate this behavior and perceive it as socially acceptable. Excessive parental phone use predicts both unresponsive parenting and elevated child externalizing problems (Teicher et al., 2010). Chronic phubbing may condition children to associate media use with attention-seeking. Meta-analyses confirm that parental phubbing significantly predicts adolescent excessive or problematic media use (Niu et al., 2020; Xie et al., 2019). On the other side, phubbing elevates child anxiety, driving compensatory media self-soothing (Liang et al., 2023), which may further increase the risk of problematic media use.

At the same time, parental phubbing is also influenced by other factors. Emerging evidence suggests that suboptimal parenting practices (e.g., inconsistency, discord) predict increased parental screen time, as conflicted caregivers may engage in “digital escape” through electronic devices (Teicher et al., 2010), while co-parenting establishes emotional support systems that mitigate negative affect accumulation (Myruski et al., 2018), thereby reducing stress-induced phone dependence through shared childcare and dyadic regulation. In addition, high-quality and harmonious co-parenting strengthens marital satisfaction and parent–child relationships while fostering a supportive family atmosphere, reducing preschoolers’ problem behaviors (Z. Wang & Cheng, 2014), potentially mitigating parental phubbing. Family systems theory conceptualizes families as dynamic, interdependent systems where behavioral patterns, rules, and roles are reciprocally influenced among all members (Bowen, 1993). This perspective highlights the importance of examining not only individual parenting behaviors, but also the interactions between subsystems—such as co-parenting—in shaping child development. Consequently, all interactions within marital and parent–child subsystems carry systemic repercussions. Thus, co-parenting families demonstrate shared childcare responsibilities and consistent rule-setting, which reduces individual stress through distributed parenting loads (Feinberg, 2003), and, consequently, reduces compensatory phubbing behaviors (Teicher et al., 2010). This evidence positions phubbing as a pivotal mediator explaining variance in the co-parenting–problematic media use linkage.

### 1.3. The Moderating Role of Secure Attachment

Importantly, the associations between co-parenting and preschoolers’ problematic media use, and parental phubbing and problematic media use may be moderated by child attachment security. Children with higher levels of secure attachment tend to exhibit greater life satisfaction, as individuals with a secure attachment style view themselves positively and are also positively perceived by others (Yoon, 2022). Secure attachment forms a stable internal working model in early childhood through consistent caregiver interactions (Ainsworth, 1979; Belsky & Rovine, 1987) and it serves as a protective factor, enabling children to access emotional resources and employ adaptive regulation strategies that buffer against familial risks (Anis et al., 2020). According to the differential susceptibility framework (Belsky & Pluess, 2009), individual characteristics, such as secure attachment, amplify or buffer the influence of environmental factors. Secure parent–child relationships act as protective factors against adverse developmental outcomes, buffering the negative impact of parental phubbing on preschoolers’ problematic media use (Yoon, 2022). By providing a safe haven, it also equips children with resilience against environmental stressors, shaping their developmental trajectories. When facing anxiety-provoking stimuli, securely attached children preferentially seek physical proximity to caregivers, employing adaptive signaling behaviors to elicit attention. fMRI evidence also confirms that attachment bonds attenuate neural responses to social exclusion, buffering the distress of rejection (Karremans et al., 2011), which demonstrates secure attachment’s capacity to buffer against adverse parental influences. In addition, the family could serve as the primary context for attachment patterns and emotion regulation strategies, shaping children’s relationships with others and their future social interactions (Wu, 2022), and individual regulatory traits can attenuate the impact of parental phubbing (X. Liu et al., 2024). Secure attachment significantly enhances positive family influences by amplifying the protective effects of cooperative co-parenting; it enables children to internalize consistent media rules, strengthens family cohesion, and fosters adaptive behaviors that reduce reliance on digital devices (Mares et al., 2018), thereby bolstering co-parenting’s role in mitigating digital risks.

Thus, secure attachment likely serves as a protective moderator, dampening risk factors for problematic media use: secure attachment moderates the influence of co-parenting and parental phubbing on preschoolers’ problematic media use by buffering negative effects and enhancing positive family influences. It stabilizes emotional responses to poor co-parenting and mitigates phubbing’s impact, reducing media reliance, while amplifying high-quality co-parenting’s benefits by fostering rule adherence and supportive family atmospheres.

### 1.4. The Present Study

**Figure 1:**
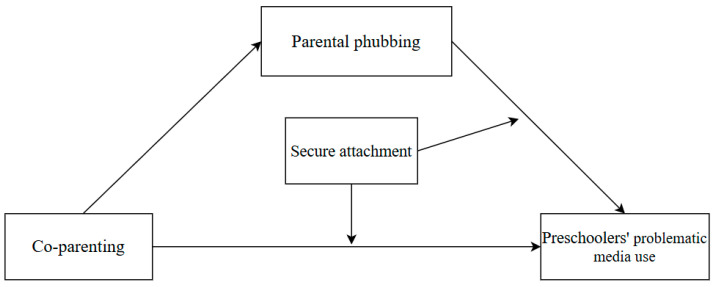
Research model.

To elucidate, the direct effects of co-parenting on preschoolers’ problematic media use and the underlying mechanism remain unexplored. As problematic media use now manifests behavioral consequences at progressively younger ages (C. Li et al., 2020), it is of significance to examine this issue among preschool populations. Thus, this study established a moderated mediation structural model (see Figure 1 for the conceptual diagram), to examine the relation between co-parenting and preschoolers’ problematic media use and the underlying mechanism. The model hypothesizes that (H1) co-parenting directly negatively predicts preschoolers’ problematic media use; (H2) parental phubbing partially mediates the relationship between co-parenting and preschoolers’ problematic media use; (H3) and secure attachment moderates both the direct path (co-parenting to preschoolers’ problematic media use) and the indirect path (parental phubbing to preschoolers’ problematic media use). Building on the theoretical framework and proposed model, this study employs a survey-based approach to empirically test the relationships among co-parenting, parental phubbing, secure attachment, and preschoolers’ problematic media use, using validated scales and a moderated mediation analysis to elucidate these dynamics. Our study was conducted in central China, a region characterized by rapid digital adoption alongside enduring traditional family norms (Yang et al., 2023). This sociocultural context provides a meaningful backdrop for examining how parenting dynamics shape preschoolers’ digital behaviors in the early developmental stage. This focus extends prior research, which often targets adolescents or older children, to a younger population where early interventions can mitigate digital risks and promote healthy development. In addition, these findings may provide evidence-based guidance for parental practices in the digital era.

## 2. Methods

### 2.1. Participants and Procedure

In total, 758 parents of preschool-aged children were recruited in central China to participate in this survey. Finally, a total of 610 valid questionnaires were obtained, resulting in an effective response rate of approximately 80.47%. The age range for fathers was 25 to 52 years (*M* = 35.83, *SD* = 4.56), while the age range for mothers was 24 to 50 years (*M* = 34.61, *SD* = 4.39). The children’s age ranged from 2 to 6 years (*M* = 4.14, *SD* = 1.17), with 319 boys and 291 girls, of which 51.8% were only children. Questionnaires were distributed using a combination of paper-based surveys, administered during parent meetings or pick-up times, and the Chinese online platform *Sojump*. All participants provided informed consent prior to participation, and a small amount of compensation was provided upon completion to encourage participation. The questionnaire survey was approved by the Ethics Review Committee of the affiliated institution, ensuring full protection of participants’ privacy and rights.

### 2.2. Measurements

#### 2.2.1. Co-Parenting

The “Co-Parenting Scale”, developed by McHale and revised by Chinese scholars in Chinese participants (C. Liu et al., 2014) was used in this study, which consists of 14 items and can be divided into two dimensions: parental support for co-parenting and parental lack of support for co-parenting. The participants were asked to respond on a 5-point Likert scale, with higher scores indicating higher levels of parental support for co-parenting. In this study, the overall reliability of the scale was 0.90, with the reliability for the supportive co-parenting dimension being 0.90 and the non-supportive co-parenting dimension being 0.84.

#### 2.2.2. Parental Phubbing

The Chinese version of “Parental Phubbing Scale” (Niu et al., 2020) was used in this study. The original scale was designed for adolescents to report on their parents’ phubbing behavior using a third-person perspective (“parents”). To align with our study’s focus on parents of preschoolers as respondents, we adapted the scale to a first-person perspective (“I”) for parental self-reports, ensuring accurate measurement of individual phubbing behaviors. The scale consists of 9 items, where parents are asked to rate on a 5-point Likert scale, ranging from “never” to “always,” based on their actual experiences. Higher average scores on the scale indicate more frequent parental phubbing behaviors. Confirmatory factor analysis indicated good validity for the scale: *χ^2^/df* = 3.15, *p* < 0.001, GFI = 0.97, TLI = 0.94, RMSEA = 0.06. Additionally, Cronbach’s alpha for this scale in the current study was 0.73.

#### 2.2.3. Problematic Media Use

The degree of problematic media use was measured using the “Children’s Electronic Media Use Questionnaire” (P. G. Li, 2018). To improve comprehension and applicability, we revised the question format into concise declarative statements. The questionnaire consists of 20 items and four dimensions: time management, interpersonal and health issues, life conflicts, and emotional experiences. Parents rated each item on a 5-point scale (1 = Hardly ever to 5 = Always). Higher total scores indicate greater levels of electronic media use in children. Confirmatory factor analysis showed good validity for the questionnaire: *χ^2^/df* = 5.87, *p* < 0.001, GFI = 0.85, CFI = 0.92, RMSEA = 0.08. In this study, Cronbach’s alpha for the dimensions ranged from 0.84 to 0.90, with the overall Cronbach’s alpha for the questionnaire being 0.96.

#### 2.2.4. Secure Attachment

This study used the Chinese version of the secure attachment dimension from the Waters Attachment Q-sort (Waters, 1995), as validated by Y. Wang et al. (2022). The scale consists of 18 items, all designed to measure secure attachment. Parents rated each item on a 5-point scale (1 = Does not apply at all to 5 = Fully applies) based on their actual interactions with their child. Higher total scores indicate a higher level of secure attachment between parents and their child. In our study, the internal consistency coefficient for the scale was 0.78.

#### 2.2.5. Covariates

Correlational analysis revealed that only subjective family socioeconomic status and parental education level were significantly associated with the core variables of interest in this study (Fam et al., 2023). Consequently, these two variables were included as control variables in the regression analysis.

#### 2.2.6. Statistical Analyses

SPSS 26.0 was used to conduct tests for common method bias and correlational analysis. Mediation and moderation effects were examined using the PROCESS macro with bootstrap procedures. To evaluate the significance of conditional direct and indirect effects, we employed bootstrap confidence intervals (CIs) calculated from 5000 resamples. Effects were deemed significant when the CIs excluded zero. Additionally, all variables, excluding control variables, were standardized prior to analysis.

## 3. Results

### 3.1. Common Method Bias Analysis

Since the data in this study were all based on parents’ self-reports, common method bias may have existed. To address this, Harman’s single-factor test was conducted, and an exploratory factor analysis was performed on all items of the questionnaires. The results revealed that 10 factors had eigenvalues greater than 1, with the first factor explaining 26.10% of the variance, which is below the critical threshold of 40%. Therefore, no significant common method bias was found in this study.

### 3.2. Descriptive Statistics and Correlational Analysis

The descriptive statistics and correlational results for all variables are presented in Table 1. The correlational analysis revealed that co-parenting was significantly negatively correlated with parental phubbing and children’s problematic media use (*p* < 0.001), but positively correlated with secure attachment (*p* < 0.001). Parental phubbing was significantly positively correlated with children’s problematic media use (*p* < 0.001). Additionally, secure attachment was significantly negatively correlated with both parental phubbing and children’s problematic media use (*p* < 0.001).

### 3.3. Testing for Moderated Mediation Model

First, to reduce multicollinearity, all variables were standardized before using Model 4 of the SPSS PROCESS macro to examine the mediating role of parental phubbing in the relationship between co-parenting and preschoolers’ problematic media use. The regression analysis showed that after controlling for family SES and parental education, co-parenting significantly negatively predicted preschoolers’ problematic media use (*β* = −0.40, *t*= −10.67, *p* < 0.001), indicating a significant total effect. Moreover, when parental phubbing was included as a mediator, the direct effect of co-parenting on the preschoolers’ problematic media use remained significant (*β* = −0.33, *t* = −8.77, *p*< 0.001). Additionally, co-parenting significantly negatively predicted parental phubbing (*β* = −0.28, *t* = −7.19, *p*< 0.001), while parental phubbing significantly positively predicted preschoolers’ problematic media use (*β* = 0.24, *t* = 6.44, *p* < 0.001). The results suggest that co-parenting not only directly negatively predicts preschoolers’ problematic media use but also indirectly influences it through the mediating role of parental phubbing. This demonstrates that parental phubbing plays a partial mediating role in the relationship between co-parenting and preschoolers’ problematic media use. As shown in Table 2, the 95% bootstrap confidence interval for the mediation effect on preschoolers’ problematic media use was [−0.10, −0.04], excluding zero, with the mediation effect accounting for 17.50% of the total effect. These results confirm H1, as co-parenting negatively predicts problematic media use, and support H2, as parental phubbing significantly mediates this association.

Subsequently, Hayes Model 15 was employed to test a moderated mediation model, examining whether secure attachment moderates both the latter half of the mediation path (between parental phubbing and preschoolers’ problematic media use) and the direct path (between co-parenting and problematic media use). As shown in Table 3, the interaction term between co-parenting and secure attachment significantly predicted preschoolers’ problematic media use (*β* = 0.08, *p* < 0.05), indicating that secure attachment moderates the co-parenting–problematic media use relationship. Furthermore, the interaction between parental phubbing and secure attachment also significantly predicted problematic media use (*β* = −0.07, *p* < 0.05), suggesting that secure attachment buffers the association between parental phubbing and preschoolers’ problematic media use.

**Figure 2:**
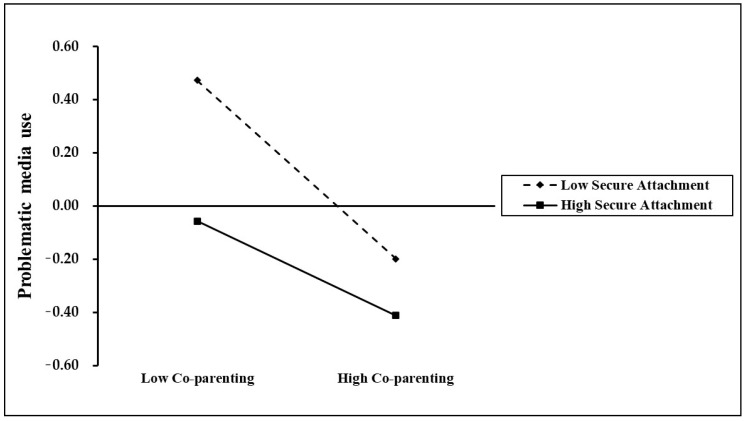
Secure attachment moderated the relationship between co-parenting and preschoolers’ problematic media use.

For a clearer interpretation of the interaction, secure attachment was split into high (+1 SD) and low (−1 SD) groups, followed by simple slope tests to assess the moderation effects. As shown in Figure 2, for the preschoolers with higher secure attachment (+1 SD), co-parenting significantly negatively predicted problematic media use (simple slope = −0.27, *p* < 0.01); whereas for those with lower secure attachment (−1 SD), co-parenting demonstrated a stronger negative predictive effect on problematic media use (simple slope = −0.52, *p* < 0.001), indicating that the negative association between co-parenting and preschoolers’ problematic media use intensifies as secure attachment levels decrease. The results indicated that the negative association between co-parenting and problematic media use was stronger among children with lower secure attachment, which supported the presence of a significant moderation effect of secure attachment.

**Figure 3:**
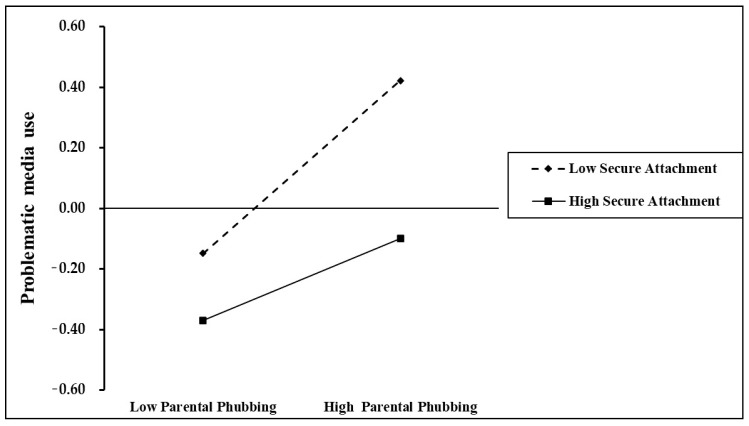
Secure attachment moderated the relationship between parental phubbing and preschoolers’ problematic media use.

Simultaneously, a simple slope analysis was conducted to examine secure attachment’s moderating role in the relationship between parental phubbing and preschoolers’ problematic media use. As illustrated in Figure 3, parental phubbing significantly positively predicted the preschoolers’ problematic media use with high secure attachment (simple slope = 0.23, *p* < 0.001); whereas the positive predictive effect was stronger for the low-secure attachment preschoolers (simple slope = 0.49, *p* < 0.001), indicating that parental phubbing exerts a greater positive influence on problematic media use for preschoolers with lower versus higher secure attachment. The stronger association in the low-attachment group indicated that insecurely attached children were more adversely affected by parental phubbing. This pattern further supported the moderating effect of secure attachment, highlighting its buffering role against parental phubbing. And these findings further supported H3, confirming secure attachment’s role in mitigating the adverse effects of poor co-parenting and parental phubbing, aligning with attachment theory’s protective mechanism.

## 4. Discussion

This study constructs a moderated mediation model examining how co-parenting influences preschoolers’ problematic media use through parental phubbing, with secure attachment moderating both the direct and indirect pathways. Based on family systems theory, social learning theory, and attachment theory, the results demonstrated that co-parenting negatively predicted the preschoolers’ problematic media use, with parental phubbing partially mediating this association. Furthermore, secure attachment moderated both the direct relationship between co-parenting and problematic media use and the indirect pathway from parental phubbing to problematic media use, with more pronounced effects observed in children with lower levels of secure attachment. These results extend previous research by clarifying the multiple mechanisms underlying preschoolers’ media behaviors, providing new insights into family dynamics in the digital era, and contribute to the literature by integrating family-level dynamics and individual child characteristics within a unified model of digital behavior development. Below, we will discuss the mediating role of parental phubbing and the moderating effect of secure attachment, highlighting both the theoretical and practical implications.

### 4.1. Co-Parenting and Preschoolers’ Problematic Media Use

Our findings revealed that co-parenting negatively predicted children’s problematic media use, which is consistent with previous research emphasizing the importance of coordinated parenting in early digital socialization (Mares et al., 2018; Sun, 2023). By establishing unified parenting rules (e.g., setting reasonable screen time limits), co-parenting reduces conflict-prone situations of children’s electronic device exposure. This consistency provides children with a sense of predictability and stability, thereby lowering the likelihood of excessive or dysregulated media use. Meanwhile, high-quality co-parenting enhances children’s emotional security, thereby decreasing their need to seek emotional compensation through media (Mares et al., 2018). As one of the most crucial factors influencing child development, effective parental communication and a harmonious family environment are typically associated with more high-quality parent–child interactions, which directly reduces children’s exposure to electronic media (Nathanson, 2015). In contrast, frequent parental conflicts drain children’s emotional resources, leaving them with limited capacity to cope with other challenges. For preschoolers in particular, exposure to conflicting parenting cues may be especially destabilizing. Children in such environments are often described as emotionally hypervigilant—constantly scanning for threats—which may disrupt their attention and emotional regulation capacities (“startled birds”), potentially leading to disengagement from daily learning and activities. This emotional burden may lead them to disengage from daily learning activities and turn to screen-based content as an accessible and predictable emotional refuge (Sun, 2023). In particular, when parents’ rules regarding children’s electronic media use conflict, children tend to shift more attention to digital devices (V. W.-P. Lee et al., 2021; Sun, 2023). Thus, our findings highlight that beyond behavioral supervision, the emotional climate shaped by co-parenting quality plays a critical role in mitigating problematic media use during early childhood.

### 4.2. Parental Phubbing as a Mediator

Moreover, the findings confirmed that parental phubbing plays a partial mediating role between co-parenting and preschoolers’ problematic media use. This finding is consistent with family systems theory (Bowen, 1993), which posits that family subsystems (e.g., marital and parent–child interactions) mutually influence family relationship, and the development of children. High-quality co-parenting, characterized by cooperation and consistency, alleviates parental stress and emotional isolation, thereby decreasing reliance on smartphones as a form of emotional escape or coping (Karremans et al., 2011; Liang et al., 2023). When parents feel supported and experience a sense of accomplishment in shared parenting responsibilities, they may be less inclined to engage in electronic devices during family interactions (Trofholz et al., 2017). Conversely, discordant co-parenting may elevate family tension and psychological disengagement, increasing parents’ tendency to use smartphones to avoid stress, which models problematic media use for children. Social learning theory further explains this mediating mechanism: children internalize problematic media use as normative through observing and imitating their parents’ smartphone overuse (H. E. Lee et al., 2022; Niu et al., 2020). Moreover, tense family environments can lead children to experience heightened stress and develop maladaptive or problematic behaviors (Krauss et al., 2020). Consequently, conflicts in parenting styles or rules may induce parental stress or disengagement, increasing phubbing behavior, which reduces parent–child interaction and media oversight, thereby elevating children’s excessive media (McDaniel & Radesky, 2018; Shao et al., 2022). This pattern could eventually manifest as emotional deficits or affective blunting and enrich family systems theory by positioning parental phubbing as a disruptive subsystem transmitting the effects of co-parenting.

The mediating role of parental phubbing thus represents a critical behavioral transmission pathway within the family digital environment. Parental phubbing not only disrupts parent–child interactions and erodes emotional intimacy, but also triggers children’s compensatory media use for attention-seeking or self-soothing (Xie et al., 2019; Yang et al., 2023). This cycle is particularly pronounced in preschoolers due to their limited self-regulation abilities and heightened susceptibility to environmental cues. This finding reveals a critical behavioral transmission pathway in family digital environments and underscores the necessity of intervening in parental media habits—particularly in families with inconsistent co-parenting—to disrupt this pathway.

### 4.3. Secure Attachment as a Moderator

Secure attachment significantly moderated both the direct relationship between co-parenting and problematic media use, as well as the latter half of the indirect pathway from parental phubbing to problematic media use. Specifically, the protective effect of co-parenting on reducing problematic media use was stronger among children with lower levels of secure attachment. Similarly, the indirect effect of co-parenting—via parental phubbing—was more pronounced among less securely attached children. These findings support the differential susceptibility hypothesis, which posits that securely attached children can utilize emotional resources to buffer environmental risks, whereas insecurely attached children are more vulnerable to adverse influences (Bowlby, 1969; Karremans et al., 2011).

In the direct pathway, secure attachment attenuated the negative association between co-parenting and problematic media use. Securely attached children typically possess more adaptive emotion regulation skills and a stable internal working model of caregiver availability, allowing them to better manage stress in the face of suboptimal co-parenting (Anis et al., 2020). Conversely, children with lower attachment security lack these internal resources and are more vulnerable to inconsistent parenting, increasing their risk of turning to digital media as a maladaptive coping strategy. This pattern aligns with previous findings that secure attachment buffers the impact of family conflict on children’s psychological adjustment (Camisasca et al., 2019). This may stem from children’s attributional patterns regarding parental phubbing: securely attached preschoolers may attribute phubbing to external factors (e.g., work demands), whereas insecurely attached children tend toward irrational attributions (e.g., “My parents don’t love me”). Interestingly, our results did not show that high secure attachment enhanced the protective effect of co-parenting. One possible explanation is that securely attached children already rely less on external environmental regulation, such as parental consistency, due to their internal emotional security. As a result, the incremental benefits of high-quality co-parenting on media regulation may be less pronounced in this group.

Similarly, secure attachment moderated the relationship between parental phubbing and preschoolers’ problematic media use, with weaker effects observed in high-secure children (Zhang et al., 2022). Securely attached preschoolers likely cope with phubbing-induced neglect by seeking proximity to caregivers, reducing reliance on media for emotional solace. In contrast, insecurely attached preschoolers may interpret parental phubbing as personal rejection, leading to intensified self-soothing behaviors through media use and a higher risk of problematic outcomes. This moderation effect highlights the protective role of secure attachment in buffering against digital risks, particularly in family environments characterized by frequent parental distraction.

### 4.4. Limitations

Several limitations must be acknowledged. The cross-sectional design precludes causal inferences; longitudinal designs tracking these behaviors across developmental periods would clarify temporal sequences. Our exclusive reliance on parent-reported measures introduces potential social desirability bias, particularly for sensitive behaviors such as parental phubbing. Nonetheless, future studies should incorporate multi-informant data (e.g., teacher reports, partner ratings, grandparent observations) and objective measures (e.g., screen time tracking apps) to complement self-reports. Moreover, the central China cultural context may limit generalizability to individualistic societies where parenting norms differ substantially—cross-cultural replications would establish universal versus culture-specific effects (Johnson et al., 2013). Additionally, our study found that the mediation effect of parental phubbing (17.50% of the total effect) is relatively low, necessitating further exploration of potential mediating and moderating mechanisms in future research. Importantly, our duration-based media use assessment overlooks qualitative aspects (e.g., content type, co-use contexts) that may differentially impact outcomes. Future research should address these limitations while exploring practical applications of these findings in family intervention programs.

## 5. Implications

This study yields important theoretical and practical implications. Theoretically, it extends family systems theory by identifying parental phubbing as a disruptive interaction pattern within the family subsystem that mediates the association between co-parenting and preschoolers’ problematic media use. It also contributes to attachment theory by demonstrating the dual role of secure attachment in both buffering negative influences and attenuating the impact of inconsistent parenting within digital family environments. Notably, this study expands prior research, which has largely focused on adolescents, by revealing that the mediating role of phubbing emerges as early as the preschool stage of development. Practically, these findings provide significant implications for family education practices in the digital era. First, co-parenting was found to indirectly reduce preschoolers’ problematic media use by decreasing parental phubbing, suggesting that enhancing collaborative parenting could serve as an effective intervention point. Thus, we recommend improving spousal communication and establishing consistent media use rules to minimize phone interference during parenting. Second, the moderating role of secure attachment supports integrating attachment theory into parenting programs, with “parent–child interaction training” recommended to enhance parental sensitivity for insecurely attached children, especially in high-stress/media-heavy contexts. Overall, these findings inform the development of evidence-based “family digital literacy” frameworks that emphasize not only media regulation rules, but also interaction quality and parental self-regulation—providing concrete avenues for early prevention of digital overuse.

## 6. Conclusions

In summary, this study employed a moderated mediation model to explore how co-parenting and parental phubbing jointly influence preschoolers’ problematic media use, yielding two primary findings: First, co-parenting was negatively associated with problematic media use, and parental phubbing could significantly mediate this relation. Second, secure attachment significantly moderated the direct relation and mediating effect; specifically, for high-secure-attachment preschoolers, co-parenting’s protection was moderate, and for those with low secure attachment, co-parenting showed stronger benefits but parental phubbing’s harms were amplified.

## Figures and Tables

**Table 1:** Descriptive statistics and correlational analysis of variables.

Variables	*M* ± *SD*	1	2	3	4	5	6
1. SES	5.21 ± 1.65	1					
2. Parental Education	4.42 ± 1.14	0.20 ***	1				
3. Co-Parenting	3.81 ± 0.65	0.16 ***	0.11 **	1			
4. Parental Phubbing	2.52 ± 0.58	−0.10 *	−0.02	−0.29 ***	1		
5. Secure Attachment	3.83 ± 0.48	0.09 *	0.09 *	0.41 ***	−0.22 ***	1	
6. Problematic Media Use	2.04 ± 0.77	−0.12 **	0.05	−0.40 ***	0.34 ***	−0.35 ***	1

Note: N = 610, * *p* < 0.05, ** *p* < 0.01, *** *p* < 0.001.

**Table 2:** Test of mediating role of parental phubbing.

Mediating Variable	Effect	Efficiency Value	BootSE	95% Confidence Interval
Parental phubbing	indirect effect	−0.07	0.02	[−0.10, −0.04]
	direct effect	−0.33	0.04	[−0.41, −0.26]
	total effect	−0.40	0.04	[−0.47, −0.33]

**Table 3:** Tests of the moderated mediation effects.

Variables	Dependent Variable: Parental Phubbing			Dependent Variable: Problematic Media Use		
	*β*	*SE*	*t*	*β*	*SE*	*t*
SES	−0.02	0.01	−1.28	−0.04	0.02	−1.71
Parental Education	0.02	0.02	0.83	0.10	0.03	3.31 **
Co-Parenting	−0.25	0.04	−7.14 ***	−0.26	0.04	−6.53 ***
Secure Attachment				−0.19	0.04	−4.78 ***
Parental Phubbing				0.21	0.04	5.76 ***
Co-Parenting × Secure Attachment				0.08	0.04	2.16 *
Parental Phubbing × Secure Attachment				−0.07	0.03	−2.15 *
*R* ^2^	0.09			0.28		
*F*	19.30 ***			33.34 ***		

Note: N = 610, * *p* < 0.05, ** *p* < 0.01, *** *p* < 0.001.

## Data Availability

The data are available on reasonable request from the corresponding author.
